# Thyroid Cancer Imaging *In Vivo* by Targeting the Anti-Apoptotic Molecule Galectin-3

**DOI:** 10.1371/journal.pone.0003768

**Published:** 2008-11-20

**Authors:** Armando Bartolazzi, Calogero D'Alessandria, Maria Gemma Parisella, Alberto Signore, Fabrizio Del Prete, Luca Lavra, Sten Braesch-Andersen, Roberto Massari, Carlo Trotta, Alessandro Soluri, Salvatore Sciacchitano, Francesco Scopinaro

**Affiliations:** 1 Cellular and Molecular Tumor Pathology Laboratory, Cancer Center Karolinska, Karolinska Hospital, Stockholm, Sweden; 2 Pathology Research Laboratory, St. Andrea University Hospital, Rome, Italy; 3 Laboratory of Nuclear Medicine, St. Andrea University Hospital, University La Sapienza, Rome, Italy; 4 S. Pietro Fatebenefratelli Hospital – Associazione Fatebenefratelli per la Ricerca (AfaR), Rome, Italy; 5 Department of Experimental Medicine and Pathology, II Faculty of Medicine, University “La Sapienza”, Rome, Italy; 6 Mabtech Research Laboratory, Nacka, Sweden; 7 Istituto di Ingegneria Biomedica (ISIB) – National Council of Research, Sezione di Roma, Italy; Genentech, United States of America

## Abstract

**Background:**

The prevalence of thyroid nodules increases with age, average 4–7% for the U.S.A. adult population, but it is much higher (19–67%) when sub-clinical nodules are considered. About 90% of these lesions are benign and a reliable approach to their preoperative characterization is necessary. Unfortunately conventional thyroid scintigraphy does not allow the distinction among benign and malignant thyroid proliferations but it provides only functional information (*cold or hot nodules*).

The expression of the anti-apoptotic molecule galectin-3 is restricted to cancer cells and this feature has potential diagnostic and therapeutic implications. We show here the possibility to obtain thyroid cancer imaging *in vivo* by targeting galectin-3.

**Methods:**

The galectin-3 based thyroid immuno-scintigraphy uses as radiotracer a specific ^99m^Tc-radiolabeled mAb. A position-sensitive high-resolution mini-gamma camera was used as imaging capture device. Human galectin-3 positive thyroid cancer xenografts (ARO) and galectin-3 knockout tumors were used as targets in different experiments *in vivo*. 38 mice with tumor mass of about 1 gm were injected in the tail vein with 100 µCi of ^99m^Tc-labeled mAb to galectin-3 (30 µg protein/in 100 µl saline solution). Tumor images were acquired at 1 hr, 3 hrs, 6 hrs, 9 hrs and 24 hrs post injection by using the mini-gamma camera.

**Findings:**

Results from different consecutive experiments show an optimal visualization of thyroid cancer xenografts between 6 and 9 hours from injection of the radiotracer. Galectin-3 negative tumors were not detected at all. At 6 hrs post-injection galectin-3 expressing tumors were correctly visualized, while the whole-body activity had essentially cleared.

**Conclusions:**

These results demonstrate the possibility to distinguish preoperatively benign from malignant thyroid nodules by using a specific galectin-3 radio-immunotargeting. *In vivo* imaging of thyroid cancer may allow a better selection of patients referred to surgery. The possibility to apply this method for imaging and treatment of other galectin-3 expressing tumors is also discussed.

## Introduction

The high prevalence of benign thyroid nodules in adult population makes the preoperative detection of thyroid cancer comparable to ‘looking for a needle in a haystack’. The prevalence of thyroid nodules increases with age, average 4–7% for the U.S.A. adult population [1] but it is much higher (19–67%) when sub-clinical nodules are also considered [2]. Fortunately, about 90% of these lesions are benign and for this reason a reliable and systematic approach to their preoperative characterization is necessary [1], [3].

The expression of Sodium Iodide Symporter (NIS) on the membrane of the thyroid cells allows the thyroid gland to concentrate iodide from the serum. NIS-mediated iodide uptake is required for the subsequent organification and oxidation steps, which are key events for the production of thyroid hormones. This peculiar property of the gland supports conventional thyroid scintigraphy, which uses radioiodine in defining the thyroid gland in both physiological and pathological states [4]. However this widely used technique does not allow the distinction among benign and malignant thyroid proliferations. In fact although cancer is unusual in thyroid nodules with efficient iodide uptake (worded *hot nodules*), the large majority of thyroid proliferations that fail to concentrate iodide (*cold nodules*) are biologically benign [4].

Normally thyroid cells do not express galectin-3. A forced expression of galectin-3 *via* specific cDNA transfection generates a transformed phenotype, blocking the apoptotic program, a feature that favors the development of cancer [5]–[9]. Interestingly, as previously reported in a large multicentre retrospective study on histological material, well-differentiated thyroid carcinomas almost invariably express galectin-3 (>94% of all thyroid cancer types, with exclusion of the medullary carcinoma), while benign thyroid proliferations do not (only 2% of the benign nodules, mostly represented by adenomas, were galectin-3 positive) [10]. This finding was confirmed by several studies reported in the literature [11]–[14] and galectin-3 immunostaining is already used in the clinical practice, at immuno-cytological level, for a better selection of patients referred to thyroidectomy [10], [15]–[17]. In this study, by using *in vivo* and *ex vivo* experimental models of thyroid cancer we show the possibility to obtain a reliable thyroid cancer imaging *in vivo* by targeting the galectin-3 lectin molecule. This diagnostic approach may be also used for imaging different galectin-3 expressing tumors *in vivo*.

## Materials and Methods

### Cell lines and galectin-3 mRNA interference

Thyroid carcinoma cell lines ARO, kindly provided by Dr. Silvia Soddu (National Cancer Institute Regina Elena of Rome, Italy) was previously described [18]–[19]. Although the thyroid origin of ARO cells has been recently questioned [20], this galectin-3 positive cell line grows very efficiently *in vivo* and provides an useful model for setting experiments of galectin-3 immunotargeting with and without galectin-3 mRNA interference. Cells were cultured in standard conditions at 37°C and 5% CO_2_ atmosphere in RPMI-1640 medium supplemented with 2 mM glutamine, 10% FCS, penicillin and streptomycin (GIBCO BRL, Gaithersburg, MD).

For galectin-3 mRNA interference three different sequences were identified and tested as reported previously [21]. The two following sequences, which strongly and similarly down regulated galectin-3 expression in ARO cells were sub-cloned into pSUPER vector and used interchangeably (conserved motifs are underlined): 5′-GATCCCCCAACAGGAGAGTCATTGTTTTCAAGAGAAACAATGACTCTCCTGTTGTTTTTGGAAA-3′(Gal3-551, sense); 5′-AGCTTTTCCAAAAA CAACAGGAGAGT-CATTGTTTCTCTTGAAAACAATGACTCTCCTGTTGGGG-3′ (Gal3-551, antisense); GATCCCCACCTTACATGTGTAAAGGTTTCAAGAGAACCTTTACACATGTAA-GGTTTTTTGGAAA-3′ (Gal3-845, sense); and 5′-AGCTTTTCCAAAAAACCTTAC-ATGTG TAAAGGTTCTCTTGAAACCTTTACACATGTAAGGTGGG-3′ (gal3-845, antisense).

**Figure 1:**
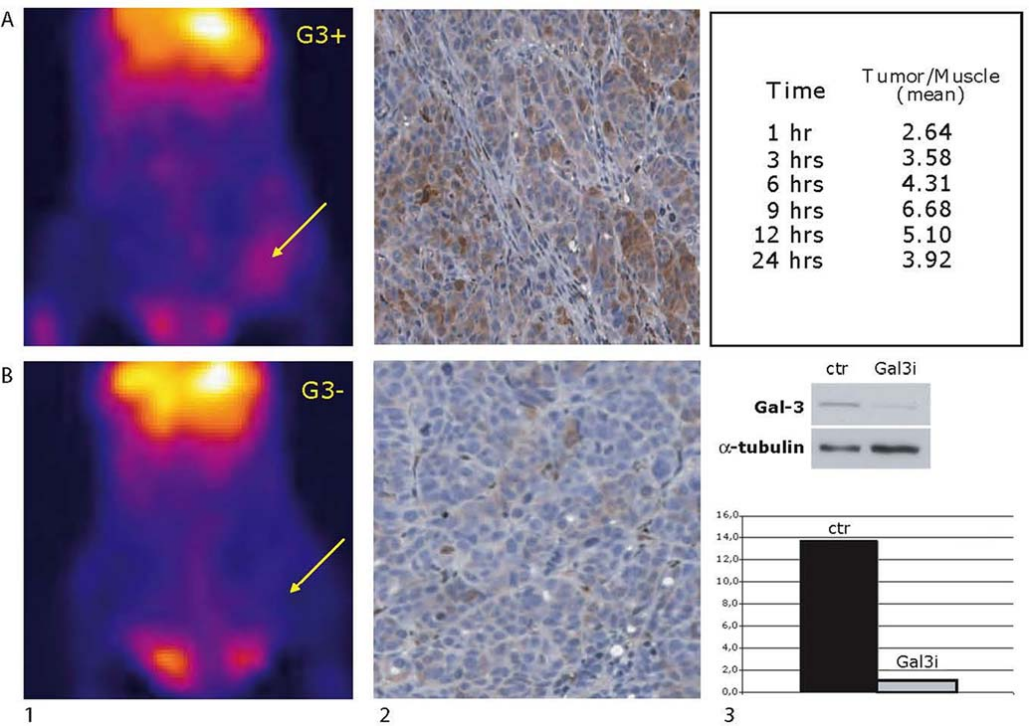
In vivo detection of galectin-3 positive ARO xenografts by using radio immunoscintigraphy with ^99m^Tc-labeled mAb to galectin-3. A) Image acquired with a high-resolution mini gamma camera in a mouse bearing ARO (Gal3+) xenograft after 6 hrs from i.v. injection of 100 µCi of ^99m^Tc-labelled mAb to Galectin-3. The arrow shows the tumor mass revealed in the left leg. A consistent accumulation of the radio tracer is observed in the liver according to the clearance of exogenous mAb (panel 1A); Morphological and immunohistochemical evaluation of the excised tumor xenograft show a poorly differentiated thyroid cancer with a variable expression of galectin-3, as revealed by a galectin-3 specific mAb and a direct immunoperoxidase staining method (panel 2A); The table shows the kinetic of tumor /normal muscle ratio of the radio tracer at different time points (panel 3A). B) Image acquired with a high-resolution mini gamma camera in a mouse bearing Galectin-3 interfered ARO xenograft (Gal3−) after 6 hrs from i.v. injection of 100 µCi of ^99m^Tc-labelled mAb to Galectin-3 (panel 1B); Immunohistochemical evaluation of the excised tumor shows a consistent down-regulation of galectin-3 expression (panel 2B); The efficiency of stable galectin-3 RNA interference in down regulating galectin-3 expression is demonstrated in immunoblotting. ARO cells mock-transfected with pSUPER vector were used as control (Ctr); galectin-3 interfered ARO cells were stable transfected with pSUPER-Gal3-551 vector (Gal3i); α-tubulin was used as a loading control. The densitometry analysis of the molecular species visualized in the gel is also shown (panel 3B). Results are expressed as relative densitometry units (rdu), measured normalizing Gal-3 signal with the corresponding α-tubulin band intensity.

In the experiment shown in **Figure 1** (panel B) ARO cells were stably transfected with pSUPER-Gal3-551 (ARO-Gal-3i) vector and mock transfected with pSUPER vector as control (ARO-ctr). Selection of stably transfected cells was performed by treatment with puromycin 2 µg/ml (Sigma) 72 hrs after transfection.

Down-regulation of Galectin-3 expression was checked in western blot analysis at different time points (12–72 hrs after transfection and tumour cells injection in mice).

### Monoclonal antibodies and immunohistochemistry

A purified horseradish-peroxidase conjugated (HRP-conjugated) rat monoclonal antibody to galectin-3 (SPACE s.r.l., Milan, Italy) was used in immunohisto-cytochemistry according to the manufacturer's instructions as previously reported [13]. Briefly, antigen-retrieval microwave treatment of tissues slides in 0.01 mol/L citrate buffer pH 6.0 was applied for three cycles of 3 minutes each at 750 W. Purified rat mAb directed to galectin-3 was used at a concentration range of 5–10 µg/ml. The enzymatic activity was visualized with 3, 3′-diamino-benzidine (Dako, Glostrup, Denmark).

### Western Blot analysis

Total cell extracts (TCEs) were obtained using a lysis buffer composed by: Tris HCl 50 mM, NaCl 150 mM, Tween 20 1%, PMSF 1 mM and 1 tablet of complete protein inhibitor cocktail (Roche). An aliquot of TCE (30–70 µg) was separated in 10% SDS-PAGE, then blotted onto nitrocellulose membrane (BIO-RAD). The following antibodies were used in immunoblotting: a purified rat mAb to galectin-3 (Mabtech AB, Nacka Strand, Sweden), a mouse mAb anti-α-tubulin (TU-02, Santa Cruz Biotechnology) and HRP-conjugated anti-mouse IgG and anti-rat IgG specific antisera (Sigma). Immunoreactivity was detected by using chemo-luminescence analysis (ECL kit, Amersham Corporation). The molecular species resolved in western blotting were finally analyzed by densitometry, using a dedicate software (NIH ImageJ, version 1.32j).

Results are expressed as relative densitometry unit (rdu) after normalization with densitometry values of the band obtained for α-tubulin.

### Murine models of thyroid cancer xenografts

Pathogen-free 4–5-week-old nude *(nu/nu)* mice (Charles River, USA) were used for establishing thyroid cancer xenografts *in vivo*. Mice were kept in cages of 4 animals each with water and food *ad libitum*. Galectin-3 expressing and highly tumorigenic poorly differentiated follicular thyroid carcinoma cell line (ARO) and the derived galectin-3 knockout ARO cells (ARO-Gal3 negative) were considered for these experiments.

Wild-type and interfered ARO cells were maintained in standard culture conditions as aforementioned, detached from tissue culture plates with trypsin 0,05%-EDTA 0,02% (Gibco), washed in sterile PBS and injected subcutaneously into nude mice at a concentration ranging from 7×10^6^ to 10^7^ cells /0.2 ml saline solution, depending by the experiment. Injection of the cells was performed by using a standard insulin needle.

No anesthesia was necessary. Fifty animals were used in three different sets of experiments for establishing tumor xenografs. Mice were examined three times a week for signs of measurable tumor growth.

Mice were selected for *in vivo* imaging experiments when tumor weight was around 0.5–1 gm. To determine the *in vivo* expression of galectin-3 in tumor xenografts, some of the tumors were surgically excised and used for galectin-3 expression analysis as reported above. This work was performed according to the specific guidelines provided by the Italian Ministry of Public Health for the animal experimentation. This study has been approved by the Institutional Ethical Committee for animal experimentation at the National Cancer Institute Regina Elena of Rome. The animal facility at the NCI of Rome is certified by the Italian Ministry of Public Health.

### Radio labeling of monoclonal antibody to Galectin-3

Affinity purified and highly concentrated (5 mg/ml) monoclonal antibody to human Galectin-3 (Mabtech, Nacka, Sweden) was radio labeled by using the method previously reported [22]. Briefly, antibody disulfide bridges were reduced using a molar excess of 2-mercaptoethanol (Sigma-Aldrich) for 30 minutes at room temperature followed by purification with a Sephadex G-25 column (Amersham Biosciences GmbH, UK) and nitrogen-purged cold phosphate buffer pH 7.4 as eluant. The reduced antibody was stored at −80°C until use. Activated galectin-3 mAb (400 µg /200 µl volume) was labeled by adding 7 µl of a methylen-diposphonate (MDP) bone scan kit (Medrotec®, Amersham Health, UK) as previously reported [22] and 10 mCi (370 MBq) of ^99m^TcO_4_^−^ freshly eluted from (^99^Mo/^99m^Tc) generator (GE-Healthcare, UK). Labeling efficiency (LE) was assessed by using Instant Thin Layer Chromatography (ITLC-SG, VWR, Italy) with 0.9% NaCl as mobile phase.

The strips were analyzed by a computerized mini-radio scanner (Bioscan System, Italy). LE, was >95% with a final specific activity of 70 µCi/µg (**Fig. S1 online**).

In order to optimize the efficiency of antibody labeling several experiments were performed varying the molar ratio 2-mercaptoethanol/mAb (range: 1.000∶1 to 4.000∶1) and the ratio mAb / ^99m^Tc for labeling (range 250.000∶1 to 4.500.000∶1).

The reduction of disulfide bridges at a molar ratio of 2145∶1 (2-mercaptoethanol /mAb) and a molar ratio of 1,500,000∶1 for Ab/^99m^Tc gave the highest LE of ^99m^Tc-anti-Galectin-3 and were used for all further experiments (**Fig. S1 online**).

Specific stability studies were performed incubating fresh radio labeled antibody in saline or human plasma at 37° C for 24 hrs and evaluating the radiopharmaceutical purity by ITLC-SG at different time points (1 hr, 3 hrs, 6 hrs and 24 hrs). The stability in saline and plasma was high during the first 6 hours (>90% and >80% for saline and plasma, respectively) with a slight decrease at 24 h (**Fig. S1 online**).

### High Resolution Gamma Camera

The High Resolution gamma Camera (HRC) (Li-tech S.r.l., Italy), shown in **figure S2 online** is composed by a crystal-collimator structure coupled to a Hamamatsu H8500 (Hamamatsu, Japan) Position Sensitive Photo Multiplier Tube (PSPMT), charge readout electronics and a data acquisition system.

The patented parallel hole collimator, already described [24]–[25] is made of pure Tungsten with 200 µm thick septa. It consists of a 24 mm collimator arranged over an adjunctive 6 mm collimator structure, in which crystals are integrated into the holes. The scintillation structure is composed by 20×20 CsI (Tl) crystals array (Spectra Physics-Hilger, UK) with a Field Of View (FOV) of 49.0×49.0 mm^2^. The crystal dimensions are 2.05×2.05×5.0 mm^3^ and each crystal is covered by100 µm white reflective epoxy on his five blind surfaces. The crystal-collimator structure is coupled to the PSPMT via optical grease.

The H8500 PSPMT has an external size of 52.0×52.0×18.0 mm^3^ with an active area of 49.0×49.0 mm^2^. The photocathode is bi-alkali and the multiplication system, composed by 12 metal channel dynodes, provides a gain of 3×10^6^ @ −1000 V.

The multiplied charge is collected by an array of 8×8 anodes.

The read-out is a miniaturized (52.0×52.0×5.0 mm^3^) front-end electronics, and the signals are sampled with a dedicated compact USB ADC board. The acquisition system provides 4 channels at 20 M Samples/sec. Data acquisition and processing is performed on a Linux Embedded System equipped with ARM CPU PXA255 with 10″ touch screen. The system control and data processing software are proprietary applications developed in C++ language. The system allows performing a real-time acquisition with a refresh time of 0.5 sec. The system is conceived to be a battery operated portable device; as a consequence the detector head weights about 2 kg while the overall weight is about 4.5 kg.

The HRC energy resolution is about 20% FWHM @ 140 keV (^99m^Tc) over the whole FOV. The sensitivity is 210 cps/MBq and the uniformity is ±5% while it provides 2.2 mm intrinsic spatial resolution suitable for our imaging experiments *in vivo* with tumour xenografted mice, and *ex-vivo* with surgical specimens derived from human patients.

### Ex-vivo imaging of human thyroid cancer

As a proof of concept for imaging differentiated thyroid cancer in human, we performed *ex-vivo* binding studies using as target a fresh metastatic cervical lymph node excised from a patient bearing a papillary thyroid carcinoma. Immediately after surgical removal the lymph node was cut in half longitudinally through the cancer lesion and the two tissue sections were incubated at 37° C for 3 h in a 50 ml solution containing 2 µCi of ^99m^Tc-anti-Gal-3 (0.1 µg protein) in saline solution with 1% HSA in presence or absence of 100 µg of unlabelled anti-Gal-3 antibody

## Results

In order to evaluate the binding specificity of a ^99m^Tc-labeled mAb to galectin-3 *in vivo*, six animals bearing galectin-3 positive carcinoma xenografts (ARO-Gal-3^+^) were considered in a preliminary experiment.

Mice with tumor mass of about 1 gm were injected in the tail vein (i.v.) with 100 µCi of ^99m^Tc-labeled mAb to galectin-3 (30 µg protein/in 100 µl saline solution). Images were acquired at 1 hr, 3 hrs, 6 hrs, 9 hrs and 24 hrs post-injection by using the mini-gamma camera. Galectin-3 immunotargeting of ARO tumors was highly efficient in xenografted mice. A central area of tumor necrosis (histologically confirmed) with impaired binding of the radiotracer was correctly depicted in one of the instances (**fig. S2 online**). Preliminary experiments with different galectin-3 positive tumors including melanomas, lymphoma and breast carcinomas were also considered for this purpose with overlapping results (data not shown). As expected, the majority of galectin-3 radio labeled antibody was concentrated in the liver according to the clearance of exogenous mAbs from the blood [26] (**fig. S2 online**). In order to optimize the imaging capture and to demonstrate the binding specificity of the galectin-3 mAb a larger *in vivo* experiment (repeated in triplicate) was considered.

The experiment included 12 xenografted mice, two groups of six animals each. The first group was transplanted with galectin-3 positive ARO cells at concentration of 5–8×10^6^ cells/0.2 ml saline solution/mouse; the second group of mice was transplanted with galectin-3 knockout ARO cells (ARO-Gal3 negative), obtained by using a specific galectin-3 RNA interference (iRNA) [20].

ARO-Gal3 negative cells were injected subcutaneously at concentration of 10^7^cells/0.2 ml saline solution/mouse, in order to promote a rapid tumor growth *in vivo* in about 4 days.

This was required for maintaining an efficient down-regulation of galectin-3 expression in stable interfered ARO cells, growing *in vivo* in absence of puromycine as selective antibiotic. Galectin-3 down-regulation in stable interfered ARO cells was checked in western blot at different time points (12–72 hrs). After 4 days from cell injection all the mice developed a visible subcutaneous tumor with a median diameter size of 0.5 cm. The animals were then injected in the tail vein with 100 µCi of ^99m^Tc-labeled mAb to galectin-3 (30 µg protein) and whole body images were acquired at 1 hr, 3 hrs, 6 hrs, 9 hrs and 24 hrs by using the high-resolution gamma camera.

Images were analyzed drawing the regions of interest (ROI) over the implanted tumor and over the counter lateral muscle taken as background. As expected the optimal visualization of the ARO-Gal-3^+^ xenografts was obtained between 6 and 9 hours from injection of the radiotracer, whereas galectin-3 negative tumors were not detected at all (**Fig. 1** panels A and B). Tumors were finally explanted for histological evaluation and immuno-phenotypical analysis as shown in **Figure 1**.

In a different set of experiments, 20 ARO xenografted mice were injected with 100 µCi of ^99m^Tc-labeled mAb to galectin-3 (30 µg protein) and 5 groups of 4 animals each were sacrificed at 3 hrs, 6 hrs, 9 hrs, 12 hrs and 24 hrs post-injection, respectively.

Tissue samples from blood, liver, spleen, kidney, small bowel, large bowel, muscle and ARO tumor xenografts were removed, weighted and counted for the presence of radioactivity. All the animals were imaged before killing with reproducible results (data not shown). The *in vivo* bio-distribution of radio labeled galectin-3 mAb expressed as percentage of injected dose per gram of tissue (%ID/g), showed a rapid blood clearance of the radiopharmaceutical within 3 hours from injection. Most of the radioactivity was detected in the liver and kidneys indicating a prompt clearance of the tracer *via* the hepatic and urinary tract (**table 1**).

**Table 1:** Biodistribution ex vivo of ^99m^Tc-labeled-mAb to galectin 3 in mice xenografted with galectin-3 positive ARO tumors.

Organ	%ID/gm				
	3 hrs	6 hrs	9 hrs	12 hrs	24 hrs
**Blood**	0.16	0.09	0.07	0.04	0.03
**Liver**	0.51	0.29	0.35	0.27	0.12
**Spleen**	0.07	0.05	0.03	0.03	0.01
**Kidney**	0.19	0.17	0.19	0.17	0.08
**Large intestine**	0.03	0.04	0.05	0.07	0.04
**Small intestine**	0.03	0.02	0.02	0.02	0.01
**Muscle**	0.01	0.001	0.001	0.001	0.001
**Tumor**	0.05	0.07	0.10	0.05	0.03

Values are expressed as % of injected dose (ID) per gram of tissue.

Five groups of mice (four animals each) bearing galectin-3 positive ARO xenografts were injected with 100 µCi of ^99m^Tc-labeled mAb to galectin-3 (30 µg protein).

Data represent the average value of %ID/gm of tissue in 4 animals sacrificed at 3 hrs, 6 hrs, 9 hrs, 12 hrs and 24 hrs post-injection of the radiotracer.

As expected the tumor uptake of the galectin-3 specific ^99m^Tc-labelled mAb increased from 3 to 9 hrs post-injection. At 6 hrs post-injection the target tumors were clearly visible while the whole-body activity had essentially cleared (**table 1** **and fig. S2 online**).

Although translation of these findings in the clinical setting will require the use of humanized mAbs linked to different radionuclides and more extensive preclinical studies, we attempted to simulate a galectin-3-based imaging of human thyroid cancer *ex-vivo*. To this aim a fresh cervical lymph node surgically excised from a patient bearing a papillary thyroid carcinoma metastasis (histologically confirmed), was used as target for studying the binding efficiency of the Gal-3 specific radiotracer.

Immediately after surgical removal the lymph node was cut in half through the cancer lesion and was incubated at 37° C for 3 hrs in 50 ml saline solution containing 2 µCi of ^99m^Tc-mAb to Galectin-3 (0.1 µg protein) and 1% human serum albumin, in presence or absence of 100 µg of unlabelled (cold) anti-galectin-3 mAb (displacement assay).

**Figure 2:**
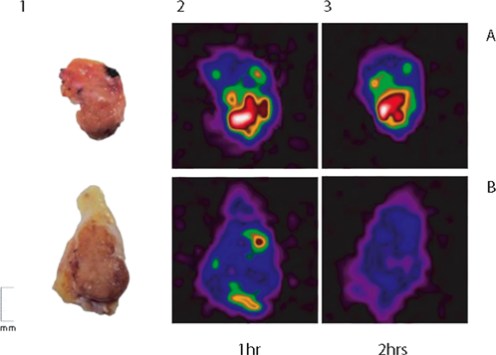
Ex-vivo binding of the ^99m^Tc-labeled mAb to Galectin-3 on papillary thyroid cancer metastasis in a lymph node. The figure shows the lymph node specimen cut in half longitudinally and imaged by using the mini gamma camera after 1 hr and 2 hrs of incubation with 2 µCi of ^99m^Tc-labeled mAb to Galectin-3 (0.1 µg protein) in saline solution, in presence (panel B) or not (panel A) of an excess (100 µg) of unlabeled galectin-3 specific mAb. Panel A shows tumour detection by radiotracer after 1 hr and 2 hrs of incubation. Panel B shows a consistent lower uptake of the radiotracer in presence of an excess of unlabelled anti-gal3- mAb, due to the specific displacement effect.

After extensive washing in saline solution a comparative imaging of the two tissue preparations was performed. Results of this experiment are shown in **figure 2**.

The mini-gamma camera promptly detected the thyroid cancer metastasis (panel A), whereas a scanty tumor uptake of the radiotracer was visible in the control, due to the displacement effect of the cold mAb in excess (panel B).

## Discussion

Altogether these results suggest the possibility to detect thyroid cancer and other galectin-3 expressing tumors *in vivo* by using a galectin-3 specific radio-immunoscintigraphy. The use of galectin-3 radiotracer for detection of thyroid cancer *in vivo* is supported by a solid molecular rationale [5], [7]–[10]. Moreover we recently demonstrated that galectin-3 immunotargeting represents an useful diagnostic tool for identifying preoperatively malignant thyroid proliferations on immuno-cytological bases, although some thyroid carcinomas (about 10–15% depending by the studies) do not express galectin-3 [10]–[14], [17]. The galectin-3 based radio-immunoscintigraphy proposed here, provides biological information about thyroid nodules and represents an useful guide to correctly identify those thyroid proliferations that should be cytologically evaluated and/or promptly excised.

Combining galectin-3 imaging with thyroid FNA-cytology, in fact, may allow to distinguishing preoperatively benign from malignant thyroid proliferations with high efficiency. As a consequence many unnecessary thyroidectomies could be avoided and the clinical use of conventional thyroid scintigraphy with radio iodide, which only provides functional information on specific thyroid conditions, could be restricted to more selected clinical questions. Further studies which use humanized galectin-3 specific mAbs conjugated to different radionuclides will be necessary to confirm and validate these findings. If the proposed diagnostic approach will prove successful a targeted radio-ablation of galectin-3 expressing tumors might also be explored by using galectin-3 specific mAbs conjugated to different radio compounds (i.e. ^186^Re, ^177^Lu or ^90^Y, ^64^Cu, ^67^Cu). A galectin-3 ‘radiation targeted therapy’ delivers the radiation dose specifically to the galectin-3 positive tumor with limited exposure of normal tissues. Penetration of beta- rays in living tissues is of several millimeters and the therapeutic effect may be obtained also in cancer cells that do not take up directly the radiopharmaceutical (cross-fire effect). This approach could be useful for both detection and treatment of micro papillary thyroid carcinomas (lesions smaller than 1 cm, commonly worded ‘*occult PTC*’), which are currently undetectable preoperatively.

Furthermore, the possibility to treat primary and metastatic thyroid malignancies that are galectin-3 positive but non-iodine avid and for this reason they are not responsive to the conventional radio-metabolic therapy with ^131^Iodine, represents an important achievement in oncology that will be further investigated.

Preliminary data of *in vivo* imaging of galectin-3 positive melanomas, lymphomas and breast carcinomas have been also obtained opening interesting possibilities for the future to use radio labeled mAbs to galectin-3 for *in vivo* imaging and treatment of different types of human malignancies [27]–[31].

## Supporting Information

Figure S1 Stability and labelling efficiency of the galectin-3 radiotracer. The stability of the radiotracer was evaluated by incubating a sample of the radio labelled mAb in saline and serum for 24 hours at 37°C. The percentage of technetium bound to the mAb was assessed by Instant Thin Layer Chromatography- Silica Gel based (ITLC-SG) strips at different time points. The graph shows retention of technetium ranging between 100% and 85% during the first six hours (around 360 minutes) with a slight decrease from 6 to 24 hours (upper panel). The activity for radiolabel mAb anti galectin-3 has been assessed in a titration experiment. The best mAb/Tc ratio has been calculated by varying the activity of 99mTc added to a stable volume of reduced antibody. The labelling efficiency (LE) was evaluated by ITLC-SG. The optimal mAb/Tc ratio found was 1.200.000/1 corresponding to 30–40 mCi of activity and a labelling efficiency up to 95%. An activity exceeding this value did not increase the LE (lower panel B).
(8.52 MB DOC)

Figure S2 Tumor imaging in vivo by using 99mTc-labelled mAb to Galectin-3 and a high-resolution portable mini gamma camera. A) The high-resolution portable mini gamma camera used in this study. B) Image captured by the high-resolution gamma camera in a mouse bearing the galectin-3 positive thyroid carcinoma xenograft ARO, after 6 hrs from i.v. injection of 100 µCi of 99mTc-labelled mAb to Galectin-3. The tumor confirmed at histology was 1.2 cm in diameter and showed a large central necrotic area corresponding to the lacuna that failed to fix the radiotracer (arrow).
(10.39 MB DOC)
