# Supplementary figures and images for: Thyroid Cancer Imaging In Vivo by Targeting the Anti-Apoptotic Molecule Galectin-3
Source: PLoS One. 2008 Nov 20;3(11):e3768. doi: 10.1371/journal.pone.0003768 (PMC2582451; doi:10.1371/journal.pone.0003768)

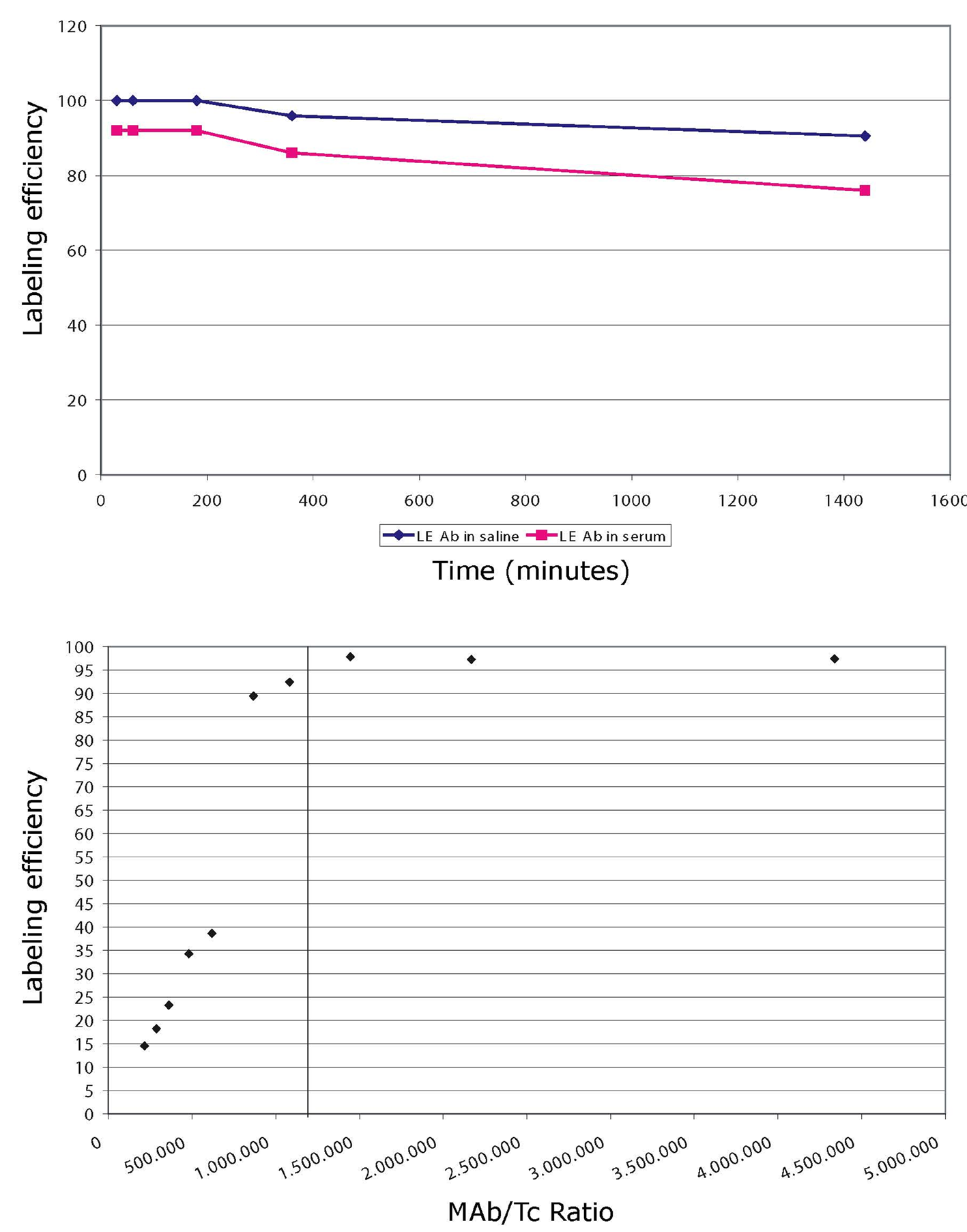

Supplement: Figure S1 — Stability and labelling efficiency of the galectin-3 radiotracer. The stability of the radiotracer was evaluated by incubating a sample of the radio labelled mAb in saline and serum for 24 hours at 37°C. The percentage of technetium bound to the mAb was assessed by Instant Thin Layer Chromatography- Silica Gel based (ITLC-SG) strips at different time points. The graph shows retention of technetium ranging between 100% and 85% during the first six hours (around 360 minutes) with a slight decrease from 6 to 24 hours (upper panel). The activity for radiolabel mAb anti galectin-3 has been assessed in a titration experiment. The best mAb/Tc ratio has been calculated by varying the activity of 99mTc added to a stable volume of reduced antibody. The labelling efficiency (LE) was evaluated by ITLC-SG. The optimal mAb/Tc ratio found was 1.200.000/1 corresponding to 30–40 mCi of activity and a labelling efficiency up to 95%. An activity exceeding this value did not increase the LE (lower panel B). (8.52 MB DOC) [file pone.0003768.s001.doc]

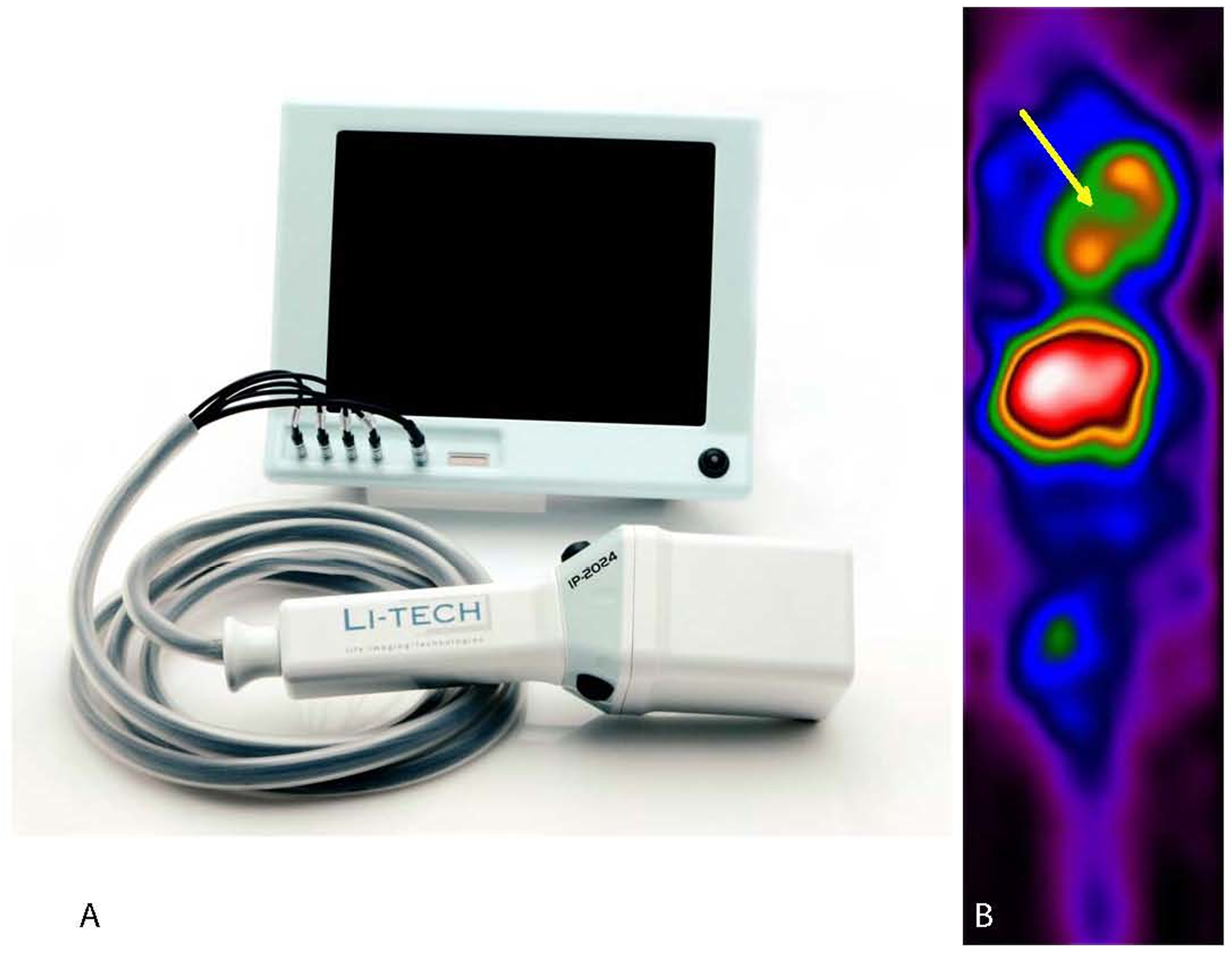

Supplement: Figure S2 — Tumor imaging in vivo by using 99mTc-labelled mAb to Galectin-3 and a high-resolution portable mini gamma camera. A) The high-resolution portable mini gamma camera used in this study. B) Image captured by the high-resolution gamma camera in a mouse bearing the galectin-3 positive thyroid carcinoma xenograft ARO, after 6 hrs from i.v. injection of 100 µCi of 99mTc-labelled mAb to Galectin-3. The tumor confirmed at histology was 1.2 cm in diameter and showed a large central necrotic area corresponding to the lacuna that failed to fix the radiotracer (arrow). (10.39 MB DOC) [file pone.0003768.s002.doc]
